# Plane heating with a transparent heater film in a fish tank

**DOI:** 10.1016/j.heliyon.2024.e24066

**Published:** 2024-01-06

**Authors:** Gustavo Panama, Juntae Jin, Dong Jin Kim, Seung S. Lee

**Affiliations:** Department of Mechanical Engineering, Korea Advanced Institute of Science and Technology, Daejeon, 34141, South Korea

**Keywords:** Plane heating, Transparent heater film, Fish tank

## Abstract

The water temperature in a fish tank is important for fish health. A conventional aquarium heater produces localized heating that causes water temperature variation, resulting in thermal stress to fish. This study presents plane heating with a transparent heater film that is aesthetically attractive when applied to fish tanks. The transparent heater film comprises a metal mesh with an optical transparency of 81 %, sheet resistance of 0.6 Ω/□, and mean heating surface temperature of 57 °C at 20 W. In the test setup, 100 W is applied to compare an aquarium heater and a transparent heater film. Increasing the water temperature from 23 °C to 24 °C at the center of the fish tank needs 28 min with the transparent heater film operating at 33 °C, whereas the same temperature increase needs 50 min with an aquarium heater operating at 49 °C. The planar heater thus results in enhanced heat diffusion and reduced water temperature variation due to its extended heating surface area.

## Graphical abstract

**Figure:**
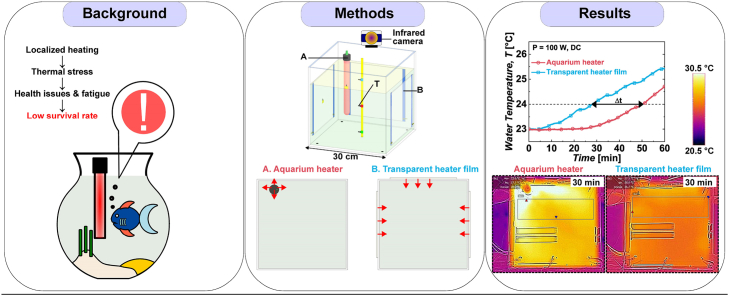

## 1 Introduction

The water temperature in a fish tank is important for fish health [1]. Intense water temperature variation can cause thermal stress and illness because most fish depend on the temperature of the aquatic environment to regulate their internal temperature [2]. Fish actively seek the optimal temperature in a thermally inconsistent environment, resulting in metabolic issues and fatigue [3]. Thus, a fish tank without a hot spot could be beneficial for the survival of animals, including pet fish.

**Fig. 1:**
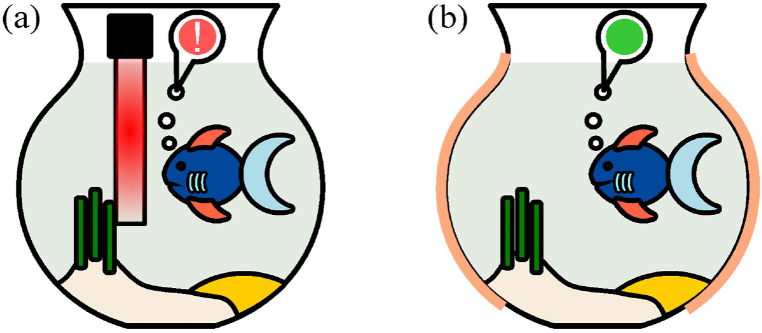
Fish tank. (a) Local heating. (b) Plane heating.

A conventional aquarium heater (AH) provides automatic control of water temperature in the range from 20 °C to 34 °C [4]. An AH operates inside the fish tank assisted by a pump to diffuse heated water. However, using agitation to assist heat diffusion compromises the device efficiency and might introduce noise-induced stress [5]. Thus, the use of AH without circulation produces localized heating shown in Fig. 1(a), which causes water temperature variation, resulting in thermal stress to pet fish.

Various plane heaters have been proposed to overcome localized water heating in a fish tank. Conductive glass walls exhibit good transparency and robust design but lack flexibility and scalability [6]. A transparent panel heater shows good flexibility, but it has low productivity [7]. An adhesive heater film is flexible and transparent, but its scalability is limited [8]. A heater film should have facile scalability and uniform heating for a variety of fish tanks.

A transparent heater film (TH) incorporates a conductive layer made of carbon, metal, oxides, etc., supported on polymer sheets [9,10]. Among these materials, metal-containing conductive layers show promise in transparent conductive film applications due to their relatively high electrical conductivity. A TH made of silver nanofiber shows low sheet resistance, but spun layers poorly adhere to plastic sheets [11]. Silver nanowire or silver nanowire composite films show good transparency and facile fabrication, but their sheet resistance is high at ∼10 Ω/□ [[12], [13], [14]]. TH can be prepared by a metal mesh with a UV embossing method, which results in good transparency, low sheet resistance, and high productivity [15].

Here, we propose a plane heating technique for fish tanks with a TH. The TH can produce a uniform thermal surface around the fish tank. The optical transparency of the TH is visually attractive for exhibition of the fish in the tank. Plane heating relies on an extended thermal surface area and lower working temperatures, as shown in Fig. 1(b).

## 2 Methodology

### 2.1 Device

**Fig. 2:**
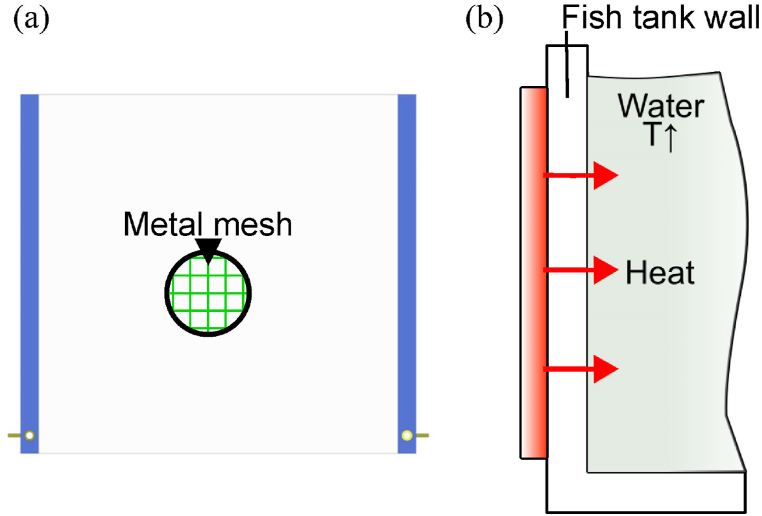
Transparent heater film in a fish tank. (a) Front view. (b) Cross-sectional view.

The TH consists of a heating surface made of a metal mesh on a polyethylene terephthalate (PET) film, as depicted in Fig. 2(a) [16]. A heating surface is desired to produce a uniform temperature field. A TH has optical transparency and enables aesthetically pleasing attachment to a fish tank wall, as shown in Fig. 2(b). THs should have higher electrical resistance than transparent electrodes to reduce the current capacity of the power supply wires [17]. Transparent electrodes require low sheet resistance to enhance electrical conductivity and reduce contact loss [18].

### 2.2 Fabrication process

**Fig. 3:**
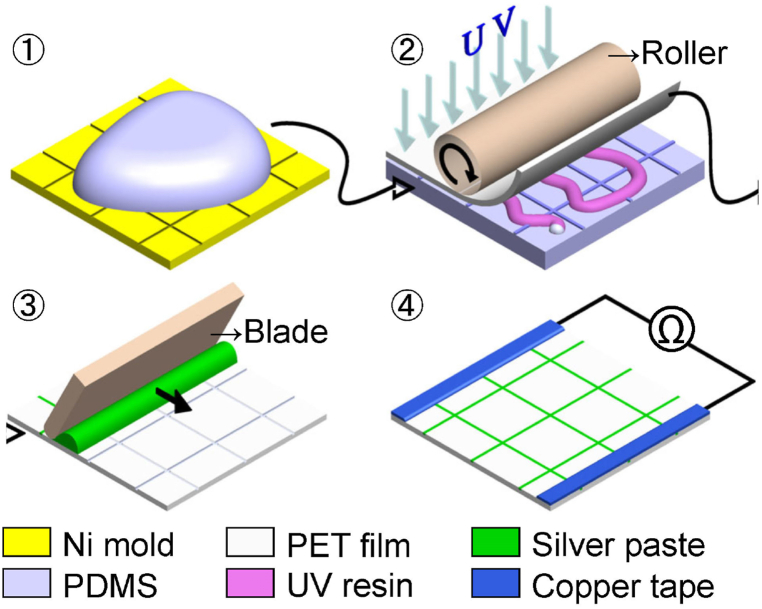
Fabrication steps of a transparent heater film: (1) PDMS mold, (2) UV embossing, (3) doctor blade, and (4) power bus.

The fabrication process of a TH is shown in Fig. 3 and includes the following steps: (1) produce of a PDMS (polydimethylsiloxane) replica from a nickel master mold, (2) transfer the patterns from a PDMS mold onto a PET substrate with UV embossing method, (3) fill in the micro trenches of the mesh pattern with silver paste by doctor blade technique, and (4) create a power bus with copper tape and coat a 4-mm-width stripe connecting the metal mesh and copper tape with silver paste [15]. The metal mesh is based on a grid design of 800-μm pitch and 10-μm linewidth. The heating surface area is 20 × 20 cm^2^, and its electrical resistance is 1.6 Ω. The electrical resistance is readily tunable by the aspect ratio or shadowing factor of the metal mesh [19,20].

### 2.3 Testing setup in a fish tank

**Fig. 4:**
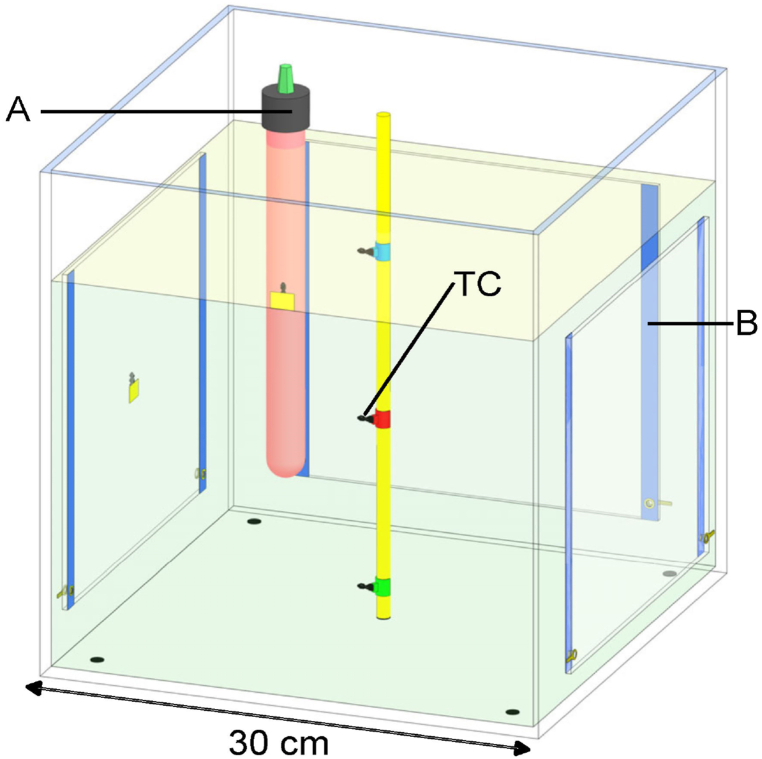
Test setup with a conventional aquarium heater (A), transparent heater film (B) and thermocouple (TC) arrangement.

The testing setup is prepared as follows: fish tank (30 × 30 × 30 cm^3^) in Fig. 4, tap water, and supplied power (100 W, DC). Both AH (Fig. 4 A) and TH (Fig. 4 B) use 100 W via ON/OFF control at a duty cycle of 70 % and a control period of 12 min. The coil resistance of AH is 440 Ω, and the resistance of 3 sheets of TH is 4.8 Ω. AH is operated inside the fish tank, and 3 sheets of TH are attached to external fish tank walls. The heating surface and water temperature at the bottom, middle and shallow levels are monitored by K-type thermocouples (Fig. 4 TC) (−267–260 °C).

The data acquisition (DAQ) platform uses a 16-channel temperature module (sensitivity: 0.02 °C), and the temperature data are saved every 370 ms. The computer-based interface allows simultaneous ON/OFF control and data processing.

## 3 Results and discussion

### 3.1 Physical properties of the transparent heater film

**Fig. 5:**
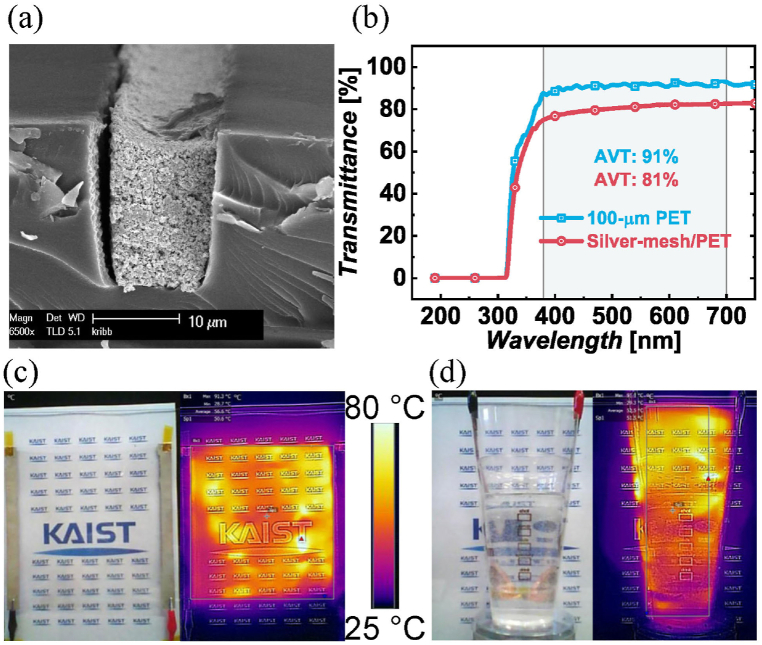
Fabrication results. (a) Scanning electron microscope image of the silver mesh line. (b) Light transmission spectrum of silver-mesh/PET film. The infrared image shows the temperature field of the transparent heater film with 20 W (c) in air and (d) attached to a plastic cup.

The thickness of the metal mesh is 17 μm, as shown in Fig. 5(a). The average visible transmittance is 81 %, which is 10 % less than that of the 100-μm PET film, as shown in Fig. 5(b) [21]. The sheet resistance (Rs) is 0.6 Ω/□. At 20 W, the average temperature in air is 57 °C, and that attached externally to the plastic cup is 52 °C, as displayed by the infrared images in Fig. 5(c)–(d). The TH is assessed based on criteria for transparent conductive films, which results in a figure of merit (FOM) of 2.8 × 10^3^, suggesting effective electrical conductivity and good transparency, as shown in Table 1 [22].

**Table 1:** Summary of transparent heater films.

Material	Method	Rs [Ω/□]	T^a^ [%]	FOM
This work	UV embossing	0.6	81	2828
Cu grid [22]	Electroplating	0.3	86	8022
AgNF [11]	Electrospinning	0.5	83	3861
AgNR [14]	Rod coating	10	98	1857
AgNR [12]	Spin coating	7.7	88	371

a Transmittance at 550 nm.

### 3.2 Water temperature monitoring with thermocouples

**Fig. 6:**
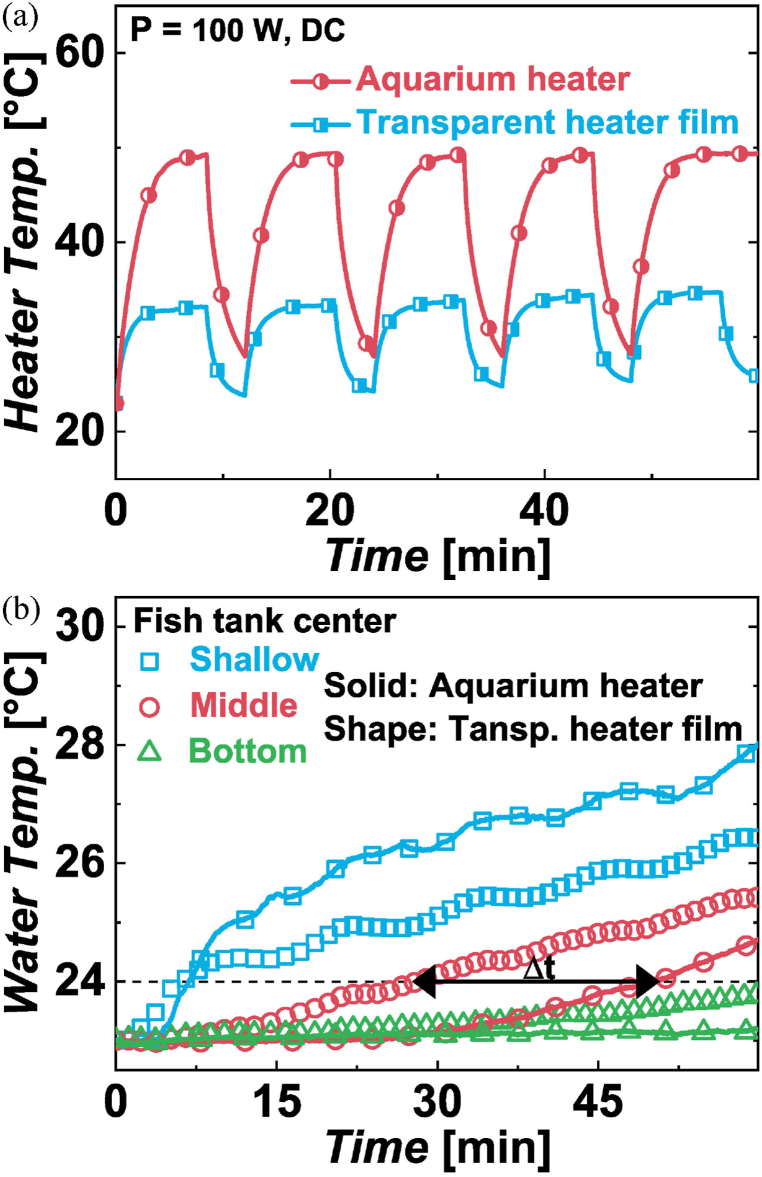
Test results. (a) Heating surface temperature of an aquarium heater and a transparent heater film at 100 W DC. (b) Water temperature with an aquarium heater and a transparent heater film.

The temperature of the heating surface or interface of an AH and a TH with 100 W is depicted in Fig. 6(a). The peak temperature with AH is 49 °C and that with TH is 33 °C. The temperature difference during ON/OFF control results in 20 °C with an AH and 10 °C with a TH. The TH produces an extended heating surface around the fish tank, whereas an AH directly exposes a hot rod with a small surface area to the aquatic environment.

**Fig. 7:**
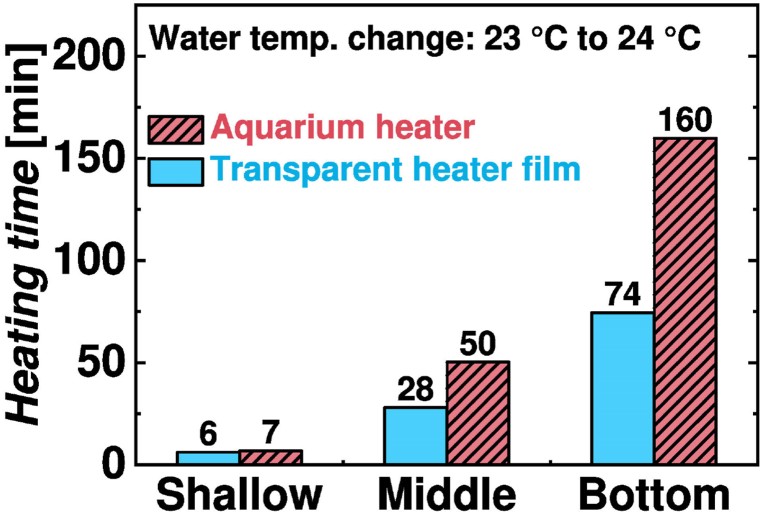
Time required to increase the water temperature from 23 °C to 24 °C at the center of the fish tank.

The water temperatures in the center of a fish tank at three different depths are shown in Fig. 6(b). The water temperature rise at the center of the fish tank begins after 6 min with a TH compared to 29 min with an AH. Additionally, Fig. 7 shows that to increase the water temperature from 23 °C to 24 °C, it takes 28 min with a TH operating at 33 °C, while it takes 50 min with an AH operating at 49 °C. After heating for 1 h, the water temperature is 25.4 °C with a TH and 24.9 °C with an AH. The heating capacity with a TH is not compromised by heat loss to the atmosphere. Faster heat diffusion with a TH results from heat transfer enhancement due to extended heating surface area.

### 3.3 Water temperature measured with an infrared camera

**Fig. 8:**
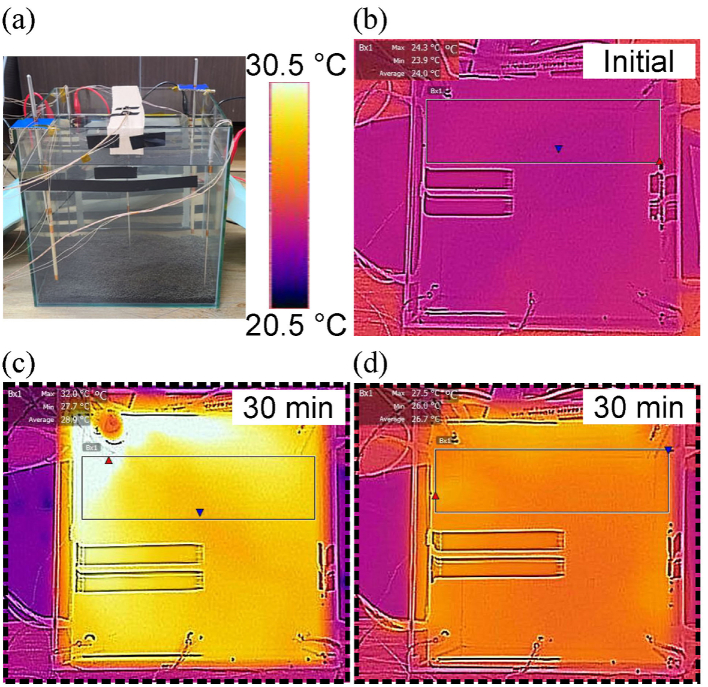
Temperature monitoring with an infrared camera. (a) Fish tank setup. (b) Temperature field of the water surface before the heating process. Temperature field of the water surface after heating for 30 min with (c) an aquarium heater and (d) a transparent heater film.

The temperature fields are obtained with a portable infrared camera (FLIR One Pro LT, -20–120 °C). The fish tank setup is shown in Fig. 8(a), where the IR camera is positioned 55 cm from the water surface. The initial thermal state is displayed in Fig. 8(b). After heating for 30 min, a large temperature gradient (between 28 and 32 °C) is caused by the AH, while a uniform temperature field (at 26 °C) with a reduced thermal gradient is produced by the TH, as displayed in Fig. 8(c)–(d).

### 3.4 Theoretical calculation of the heat transfer rate ratio

Heat transfer occurs by external convective flow with an AH and internal convective flow with a TH [23]. The heat transfer rate is described by Fourier's law of conduction for thermal conduction and Newton's law of cooling for thermal convection, as shown in Table 2 [24]. The ratio of the heat transfer rate (Q̇) between the TH and the AH is estimated based on the following information: (1) temperature difference during ON/OFF control; (2) water at 23 °C, AH at 49 °C, and TH at 33 °C. Heat transfer enhancement with the TH relative to the AH corresponds to a factor of 6 for thermal conduction and a factor of 4.6 for thermal convection. These improved thermal properties are attributed to the extended heating surface area of the TH.

**Table 2:** Estimation of the relative heat transfer rate.

Principle	ΔT [°C]	Area [cm^2^]	Q̇TH/Q̇_AH_
Thermal Conduction^a^	ΔT_TH_ = 10 ΔT_AH_ = 20	A_TH_ = 1200 A_AH_ = 100	6.0
Thermal Convection^b^	ΔT_TH_ = 10 ΔT_AH_ = 26	A_TH_ = 1200 A_AH_ = 100	4.6

a Fourier's law of conduction: ${\left({\dot{Q}}_{cond}\right)}_{plane}=k_{t}A\left(\frac{T_{hot}-T_{cold}}{\Delta x}\right)$.

b Newton's law of cooling: ${\dot{Q}}_{conv}=hA\left(T_{water}-T_{heater}\right)$.

Glass has a higher thermal conductivity (κ = 1.06 W/m·K, 296 K) than water (κ = 0.61 W/m·K, 300 K) [25,26]. Thus, fish tank walls with a TH form a planar uniform thermal surface, while a hot region is produced around an AH glass housing.

## 4 Simulation

**Fig. 9:**
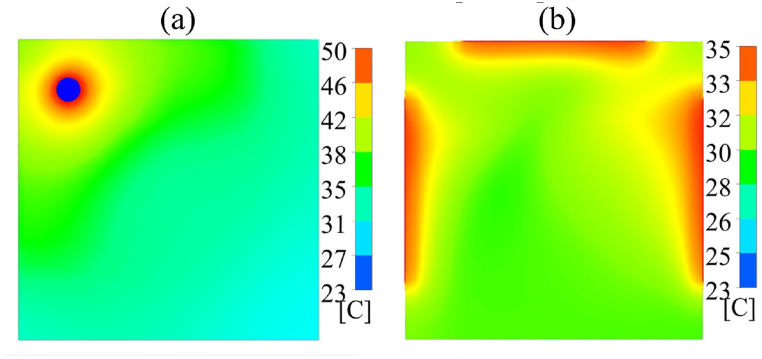
Thermal fluid simulation. Temperature field of the water surface after 3 h with (a) local heating and (b) plane heating.

The simulation is performed using the energy equation model in ANSYS-Fluent [27]. The setup model is as follows: water at 23 °C in a glass container, an AH modeled as a glass cylinder at 50 °C, a TH modeled by a metal sheet at 35 °C, and dimensions as described in Section 2. Material properties are provided by the ANSYS library. The heat transfer coefficient through the floor wall is 1000 W/m^2^K, the remaining walls are adiabatic, and the temperature of the surrounding medium is 23 °C. The simulation results of the top view show local heating with AH compared to plane heating with TH after 3 h, as displayed in Fig. 9(a) and (b). Numerical studies of water-based fluids with a plane heater and a rectangular container show evenly proportioned heat flow streamlines, which are consistent with the result with a TH [28,29].

## 5 Conclusions

Local heating produced by the conventional aquarium heater causes water temperature variation in a fish tank, which can induce thermal stress in animals including pet fish. Therefore, plane heating in a fish tank is shown here using a transparent heater film that allows for visually attractive integration with the fish tank wall, which does not compromise view of fish in the tank. The transparent heater film is based on a metal mesh with a transmittance of 81 %, sheet resistance of 0.6 Ω/□, and mean temperature of 57 °C in air with 20 W. Increasing the water temperature from 23 °C to 24 °C at the center of the fish tank takes 28 min with a transparent heater film operating at 33 °C, while it takes 50 min with an aquarium heater at 49 °C (both with 100 W). Thermal images reveal that local heating with an aquarium heater causes an intense thermal gradient, whereas plane heating with a transparent heater film produces a uniform temperature field. The heat transfer enhancement with the transparent film heater relative to the standard aquarium heater is estimated to be 6:1 for thermal conduction and 4.6:1 for thermal convection. These enhancements are attributed to the extended heating surface area of the transparent heater film. Plane heating with the transparent heater film enhances heat diffusion and reduces water temperature variation, which is beneficial to increase the survival of sensitive aquatic species. An extension of this work would be to modify the film heater into an immersible plane heater to further increase the thermal efficiency.

## Additional information

No additional information is available for this paper.

## CRediT authorship contribution statement

**Gustavo Panama:** Writing – original draft, Visualization, Validation, Software, Investigation, Formal analysis. **Juntae Jin:** Methodology, Conceptualization. **Dong Jin Kim:** Methodology, Conceptualization. **Seung S. Lee:** Writing – review & editing, Supervision, Project administration, Funding acquisition.

## Declaration of competing interest

The authors declare that they have no known competing financial interests or personal relationships that could have appeared to influence the work reported in this paper.
